# Functional analyses of heteromeric human PIEZO1 Channels

**DOI:** 10.1371/journal.pone.0207309

**Published:** 2018-11-21

**Authors:** Radhakrishnan Gnanasambandam, Chilman Bae, Lynn Ziegler, Frederick Sachs, Philip A. Gottlieb

**Affiliations:** Department of Physiology and Biophysics, State University of New York at Buffalo, Buffalo, United States of America; Indiana University School of Medicine, UNITED STATES

## Abstract

PIEZO1 and PIEZO2 are mechanosensitive channels (MSCs) important for cellular function and mutations in them lead to human disorders. We examined how *functional* heteromers form between subunits of PIEZO1 using the mutants E2117K, E2117D, and E2117A. Homomers of E2117K do not conduct. E2117A homomers have low conductance with rapid inactivation, and those of E2117D have high conductance with slow inactivation. Pairing E2117K with E2117D or E2117A with E2117D gave rise to new channel species representing heteromers with distinct conductances. Whole-cell currents from co-expression of E2117A and E2117D fit well with a linear-combination model of homomeric channel currents suggesting that functional channels do not form from freely-diffusing, randomly-mixed monomers *in-vitro*. Whole-cell current from coexpressed PIEZO1/PIEZO2 also fit as a linear combination of homomer currents. High-resolution optical images of fluorescently-tagged channels support this interpretation because coexpressed subunits segregate into discrete domains.

## Introduction

PIEZO1 and PIEZO2 are cation-selective ion channels that activate in response to mechanical stimuli and rapidly inactivate in a voltage-dependent manner [1–3]. Cryo-EM studies show that the PIEZO1 channel is a trimer (molecular mass of ~900 kD) [4–6] with a central pore formed at the interface of its subunits.

Dysfunction of PIEZO1 causes hereditary xerocytosis (HX), a hemolytic anemia of red blood cells [7], and congenital lymphatic dysplasia, whereas mutants of PIEZO2 cause muscular atrophy with perinatal respiratory distress, arthrogyposes and scoliosis [8–13]. The mutations in PIEZO1 that cause HX slow inactivation [8, 10, 14] and introduce a random latency (>100 ms) to activation [10]. We reasoned that the protein products of mutant *PIEZO1* alleles can assemble into heterotrimeric channels differing in stoichiometry (e.g., 1 or 2 similar subunits in a trimeric channel), and consequently kinetics, and may thereby modulate the phenotype of diseases such as HX.

In this work, we examined patch clamp data of the mechanical response of cells coexpressing mutant channels (E2117K: non-conducting, E2117A: low conductance and fast inactivation, E2117D: high conductance and slow inactivation). These mutant human channels are similar to those of mouse Piezo1 derived by substitutions at E2133, a residue that does not line the pore [4, 6, 15, 16]. With single-channel resolution, coexpression of pairs of different mutants gave rise to channels with new conductances that were usually intermediate to those of the homomers, which is consistent with the formation of heteromers.

We used quantitative kinetic modeling to analyze whole-cell current from cells cotransfected with a human PIEZO1 mutant pair (E2117A and E2117D) as well as that from cells cotransfected with human PIEZO1 and PIEZO2. In both cases, currents seemed to be a sum of the contributions from discrete populations of homomers, without a significant quantity of heteromers. The absence of evident heteromeric whole-cell currents suggests that the functional channels were not derived from free-floating populations of monomers, but were formed from clusters of homomeric subunits.

## Results

**Fig 1:**
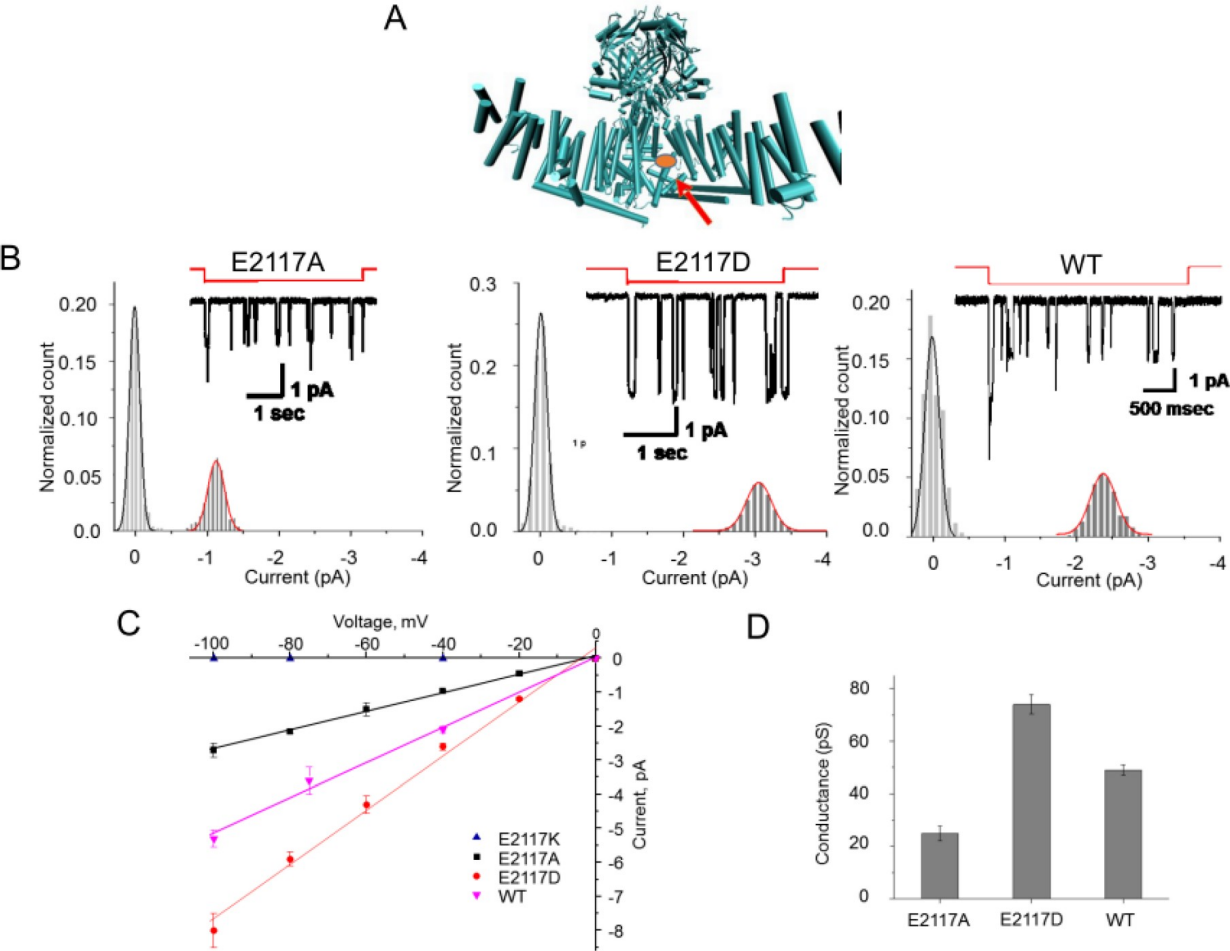
Mutations at position 2117 of hP1 altered channel conductance. **(A)** indicates the location of the 2117D (red ball and arrow) using the mouse CryoEM structure of mouse PIEZO1. **(B)** shows single channel currents from a cell-attached patch containing E2117A **(Left)**, E2117D **(Middle)** and the wild-type **(Right)**. The amplitude histograms of unitary currents are included. All recordings done in a high K^+^ bath to clamp the membrane potential to near zero. The pipette solution was 160 mM KCl without divalent ions. The suction stimulus of -20 mmHg is in red above the current trace, and the duration was 3–4 seconds at -50 mV. **(C) (Left)** shows the current-voltage relationship at negative membrane potentials. The slopes are 25 pS (R^2^>0.99) for E2117A, 73 pS (R^2^>0.99) for E2117D, and 49 pS (R^2^>0.99) for the wild-type. **(D)** the mean current, averaged over multiple patches at -100mV is summarized in the bar graph **(Right)**. In all cases, the currents reversed near 0 mV. These data are an aggregate response from > 15 patches.

Mutating the glutamic acid at position 2117 to alanine, cysteine, aspartate, lysine, or serine caused significant changes in the unitary conductance of PIEZO1 in HEK293 cells (**Fig 1 and S1 Fig**). **Fig 1A** shows the location of residue 2117 using the mouse PIEZO1 cryo-EM structure [4, 5, 16]. **Fig 1B** shows the unitary currents of two homomeric mutant channels (E2117A and E2117D) and the endogenous wild-type channel. E2117A lowered the unitary conductance of PIEZO1 from 51 ± 2 pS [17] to 25 ± 2 pS at -100mV (**Fig 1B, 1C and 1D**). At -100 mV, E2117C and E2117S had conductances of 33 ± 1 pS and 37 ± 2 pS, respectively (**S1 Fig**). We did not observe unitary currents with E2117K (n = 14), whereas unitary conductance *increased* to 73 ± 4 pS for the E2117D mutant (**Fig 1B, 1C and 1D**). **Fig 1D** summarizes these data. We noted that the data from mutants fit well to single populations of mutant channels and with no significant population of functional heteromers formed with native channels [18].

The reversal potentials for E2117A and E2117D were similar to that of the wild-type channel (**Fig 1C**). The unitary conductance of wild-type channels with Li^+^ as the charge carrier is roughly half of that with K^+^ [10]. The Li^+^ conductances of both E2117A and E2117D were lower than that with K^+^ suggesting that position 2117 is not in the selectivity filter (**S2 Fig**), consistent with the cryo-EM structure of mouse Piezo1 [4]. The selectivity for cations over anions (substituting Gluconate^-^ for Cl^-^) was also unaffected (**S2 Fig**). Neither M2225R nor R2456H, both of which are mutations associated with hereditary xerocytosis [8, 10, 14], affect the unitary conductance of PIEZO1 [10].

### Macroscopic currents from E2117A and E2117D

**Fig 2:**
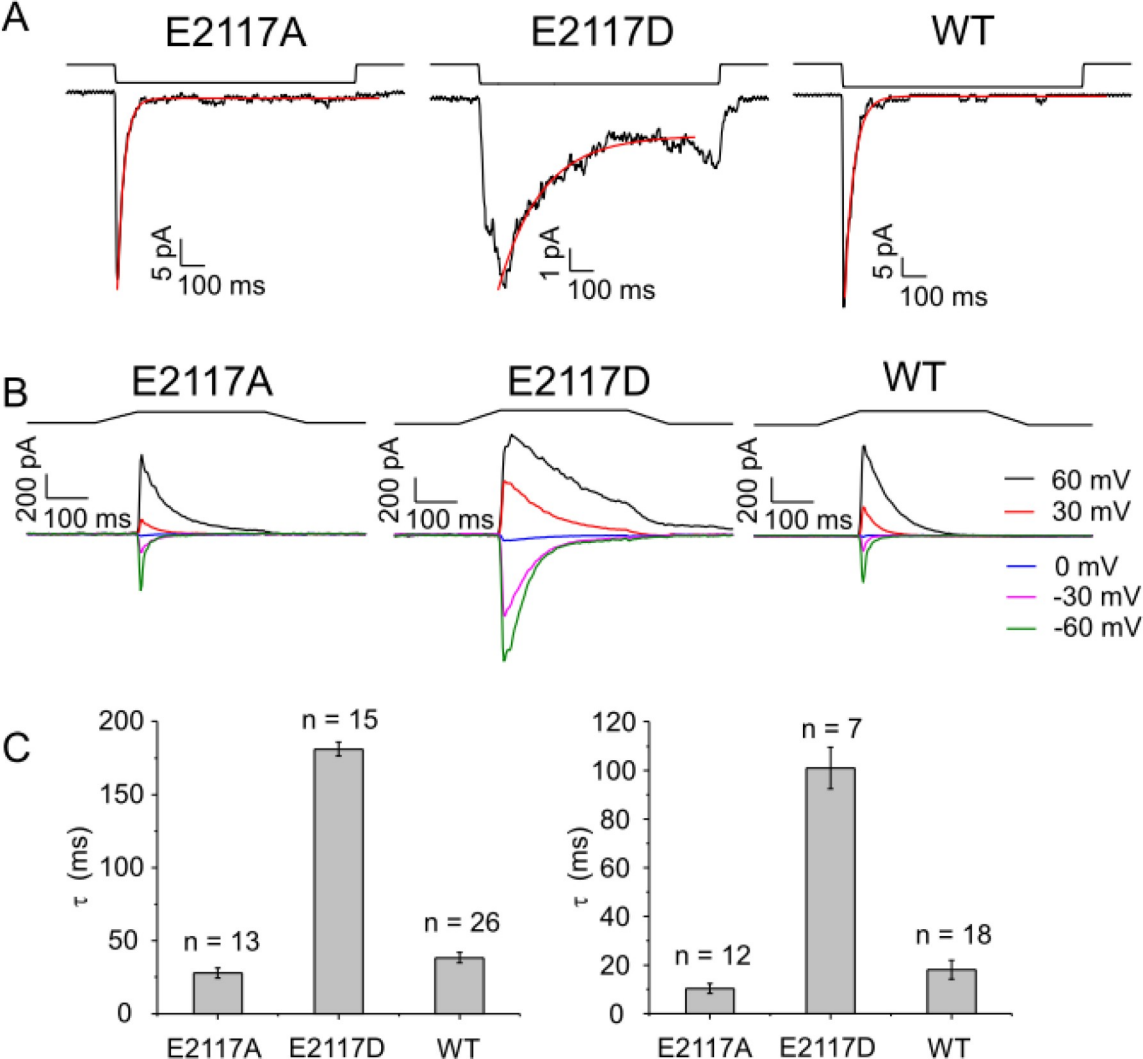
A conservative mutation of glutamic to aspartic acid slows inactivation. **(A)** shows cell-attached patch currents comparing the inactivation rates of E2117A, E2117D and wild-type. The inactivation time constant fit to a single exponential (red trace). The inactivation rate derived for E2117A **(Left)** is nearly identical to wild-type **(Right)**, but E2117D **(Middle)** is ~ 6 fold slower. **(B)** shows inactivation of whole-cell currents for the two mutants at -60 mV. The inactivation of E2117D is ~10-fold slower than E2117A. The latter’s inactivation rate is similar to that of the wild-type. **(C)** shows the inactivation time-constant averaged across multiple patches and cells. Results are summarized in the bar graph. For cell-attached patches, the inactivation time-constants for E2117A, E2117D and wild-type at– 100 mV were τ = 28.0 ± 3.4 ms, 181.3 ± 4.7 ms, and 38.4 ± 3.6 ms, respectively. For whole-cell recordings, the inactivation time constants at -60 mV for E2117A, E2117D, and wild-type were τ = 10.2 ± 1.7 ms, 101.2 ± 8.7 ms, and 17.6 ± 3.8 ms, respectively.

Both E2117A and E2117D activated rapidly resembling the wild-type in this respect [10] (**Fig 2**). E2117D inactivated slower (τ = 181.3 ± 4.7 ms) than E2117A (τ = 28.0 ± 3.4 ms) (**Fig 2A and 2C**); the inactivation rate of E2117A is comparable to that of the wild-type (τ = 38.4 ± 3.6 ms). Whole-cell inactivation rates of E2117D (τ = 101 ± 8.8 ms) and E2117A (τ = 10.2 ± 1.7 ms) resembled that of the wild-type (τ = 17 ± 4 ms at -60 mV) recorded in the cell-attached configuration (**Fig 2B and 2C**). As with the wild-type, we observed no discernable latency to activation with E2117A or E2117D [10]. This is in contrast to the xerocytosis mutants (M2225R and R2456H), both of which display marked random latencies.

**Fig 3:**
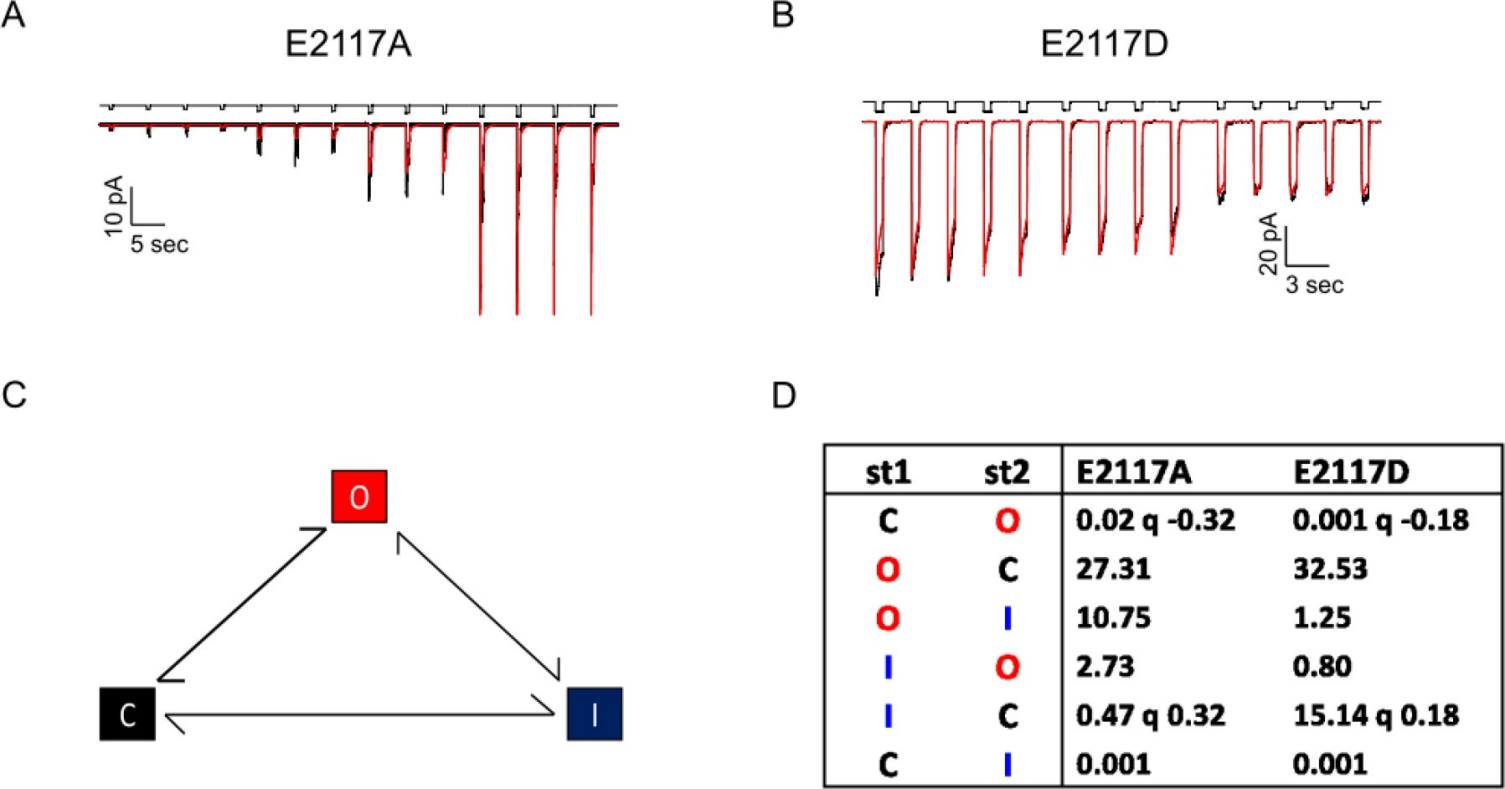
In cell-attached patches, the pressure gating sensitivities of E2117A and E2117D are different. **(A)** shows data from E2117A and **(B)** shows data from E2117D. The suction pulses are above. Each pressure pulse is repeated 5 times (-40 mmHg, -30 mmHg and -20 mmHg). In red is the fit to a 3-state closed-loop model of closed (C), open (O), inactivated (I) in detailed balance using QuB software [10] **(C)**. The transition from closed to open and inactivated to closed are pressure-dependent. **(D)** shows the rate constants (s^-1^) derived for the model, with the pressure sensitivity of the rates denoted as *q* (the rate constants are of the form k_i,j_ = k0_i,j_ exp(q_ij_*P) where P is the stimulus pressure (actually negative since the stimulus is suction—see **S3 Fig** for SD). The *q* value for E2117A (-0.32) is twice that of E2117D (-0.18), which is similar to the wild-type (-0.16)[10]. The higher value of *q* may reflect changes in the dimensions of the closed and/or open states, or changes in the local tension.

We quantified the kinetics [2] of E2117A and E2117D by fitting their cell-attached patch currents (**Fig 3A and 3B**) using the macroscopic analysis routine of QuB software (MAC) [19]. From the familiar cyclic three-state model [10] we obtained estimates of the six rate constants maintained in detailed balance [19] (**Fig 3C and 3D, and S3 Fig).** The inactivation rate of E2117D was approximately 10-fold slower than that of E2117A. The model also revealed that the pressure dependence of the activation rate of E2117A was approximately twice that of the wild-type [10] and E2117D. The slope of the pressure sensitivity curve is a measure of the dimensional changes between the closed and open states [10, 20].

### Investigation of functional heteromers

**Fig 4:**
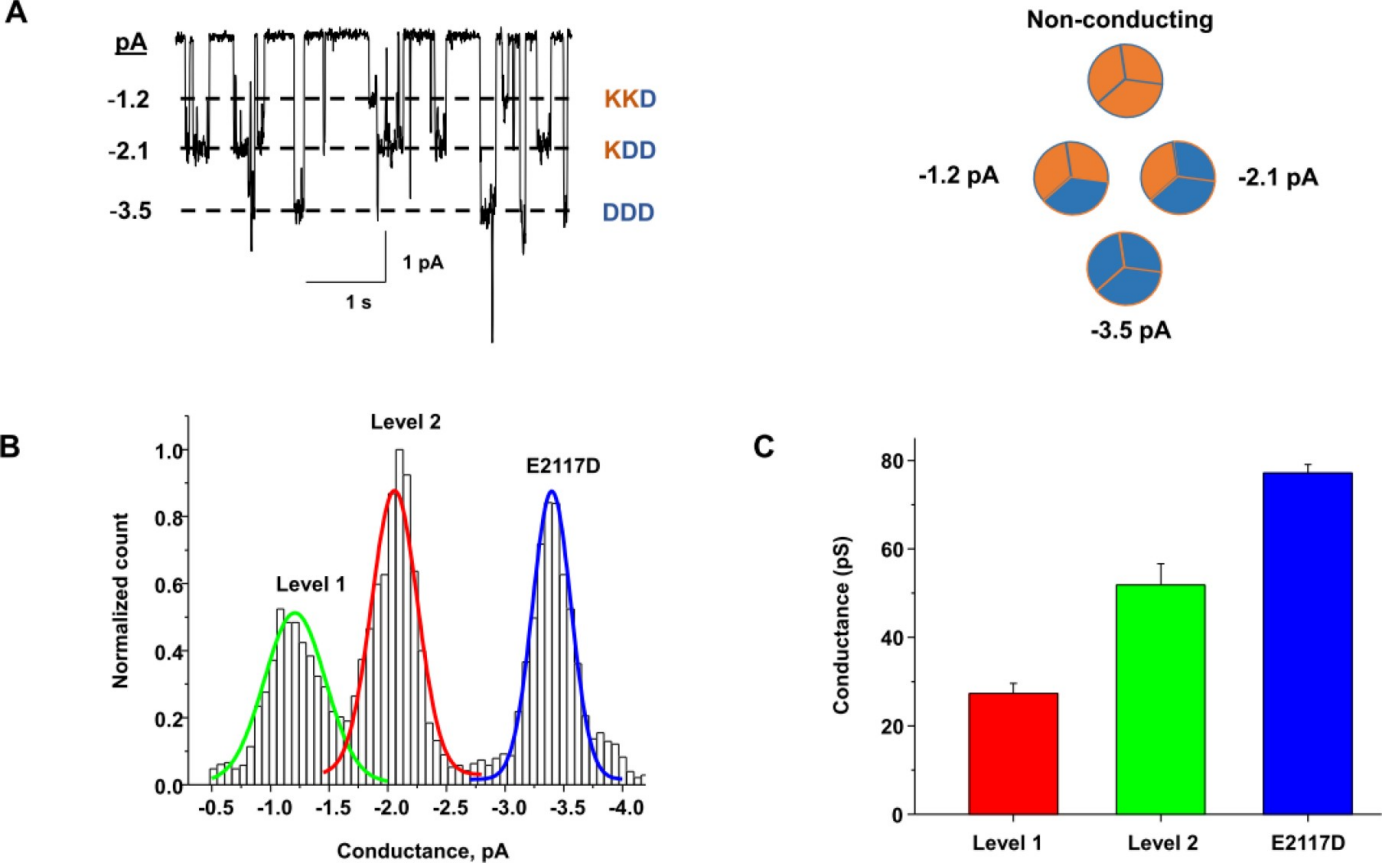
Coexpression of mutant channels forms new conductance levels. Plasmids for E2117K and E2117D were co-transfected and each contained an IE element that produced a different fluorescent marker to ensure that the cells under study expressed both proteins. We used mCherry to tag the expression of E2117D and GFP for E2117K. **(A) (Left)** is an example of single channel currents from a cell-attached patch with discrete conductance levels as indicated. The stimulus was 4 seconds at -10 mmHg. A potassium bath set the membrane potential near 0 mV and the applied membrane voltage was -50 mV. **(A) (Right)** are possible combinations of heteromers from subunits. Blue refers to E2117D and orange is E2117K. **(B)** is the amplitude histogram with three different current levels indicated. The highest level is monomeric E2117D (blue line). Only two *new levels* appeared (red and green lines). **(C)** is a bar graph of the three conductance levels (green, red, and blue). These data are the mean responses from 5 different patches with different ratios of transfected DNA. Each patch was stimulated with suction pulses ranging from -10 to -30 mmHg for 2 to 10 seconds at potentials from -50 to -100mV.

To look for functional heteromers we used single channel conductance as an indicator. We co-expressed mutants with distinctly different unit conductances and looked for new discrete unitary conductance levels that would be intermediate to those of the E2117A and E2117D homomers (other multi-subunit channels have been analyzed in this manner [21, 22]). We co-transfected E2117K and E2117D in various ratios (200–500 ng total) and looked for evidence of heteromeric channels in cell-attached patches (**Fig 4**). We cotransfected two bicistronic expression vectors (each vector expressed a channel and a fluorescent protein) to monitor and confirm expression of both channels (See Methods). **Fig 4A** shows that there were unitary current levels not seen with homomeric channels. **Fig 4B** is an amplitude histogram summarizing the data for patches from cells coexpressing E2117K and E2117D. There were three discrete current levels: -1.2 ± 0.2 pA, -2.2 ± 0.2 pA and -3.5 ± 0.2 pA at -50 mV (**Fig 4A**). The aggregate unitary conductance of level 1 was 27 ± 2.3 pS, that of level 2 was 51 ± 4.8pS, and that of the unmodified E2117D homomer was 77 ± 1.5 pS (-100 mV) (**Fig 4C**). These levels match the anticipated levels since the E2117K homomer does not conduct.

**Fig 5:**
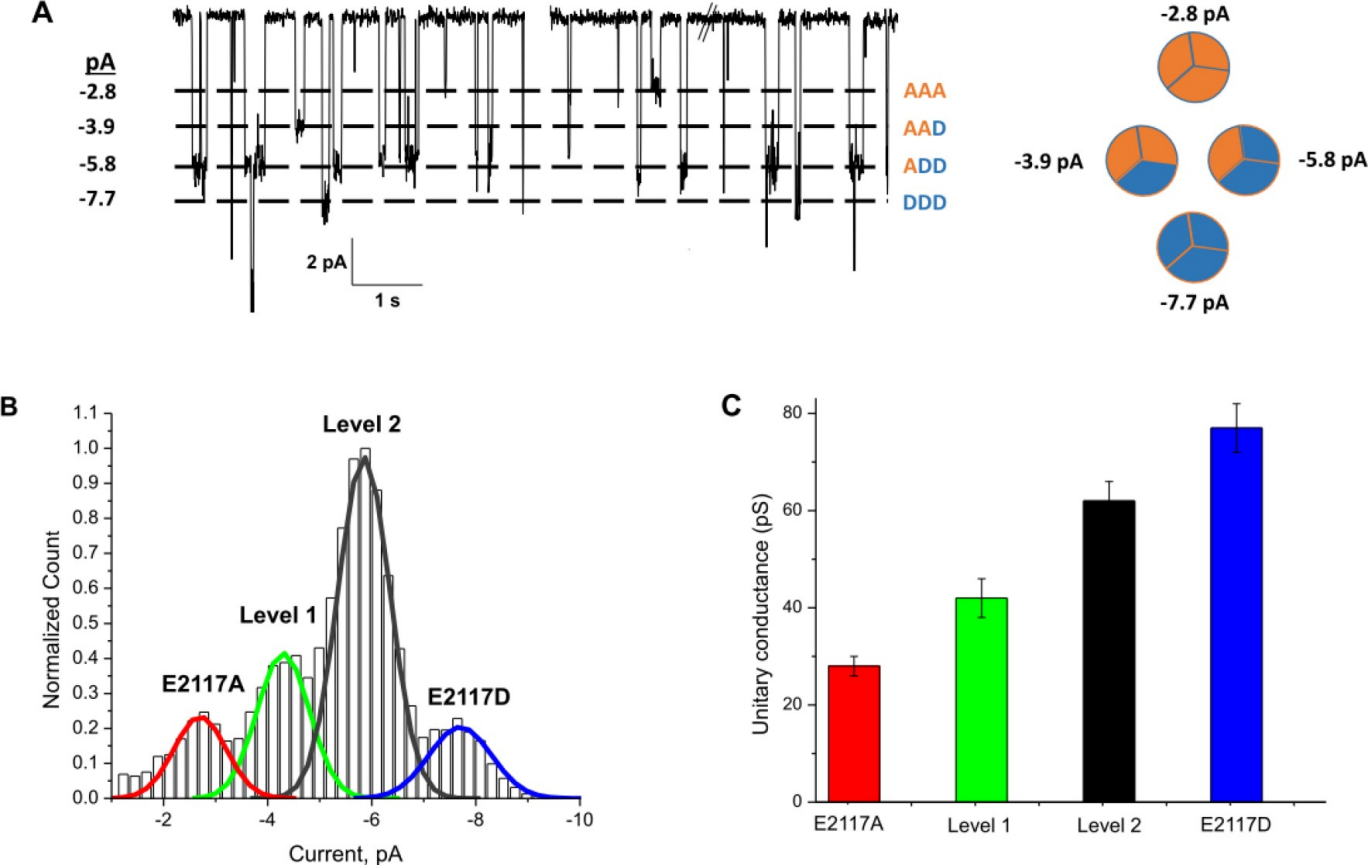
Coexpression of E2117D and E2117A mutant channels form new conducting levels. Data were collected at -100 mV with a 60 second pulse at -12 mmHg. **(A) (Left)** shows a current trace with discrete conducting levels indicated to the left of the trace. The displayed data are from a single pulse but are not contiguous. **(A) (Right)** are possible combinations of heteromers from subunits. Blue refers to E2117D and orange is E2117A. Based on the amplitudes **(B)**, the low level of ~-2.8 pA is homomeric E2117A and the current level at -7.7 pA is homomeric E2117D. The amplitude histogram in Panel B shows two new levels at -3.9 pA and -5.8 pA.

We then used the combination of E2117A and E2117D to form heteromers. We observed unitary currents whose amplitudes matched those of the homomers (-7.7 ± 0.2 pA and -2.7 ± 0.45 pA, **Fig 5A and 5B**), and importantly, we observed unitary currents of -5.8 ± 0.3 pA and -3.9 ± 0.4 pA (**Fig 5**) amplitudes. These latter two intermediate-level unitary currents are from *functional* heteromers. We observed four different conductance levels in the population (N = 10) although not all patches generated all of these unitary conductances.

### Analysis of whole-cell currents

**Fig 6:**
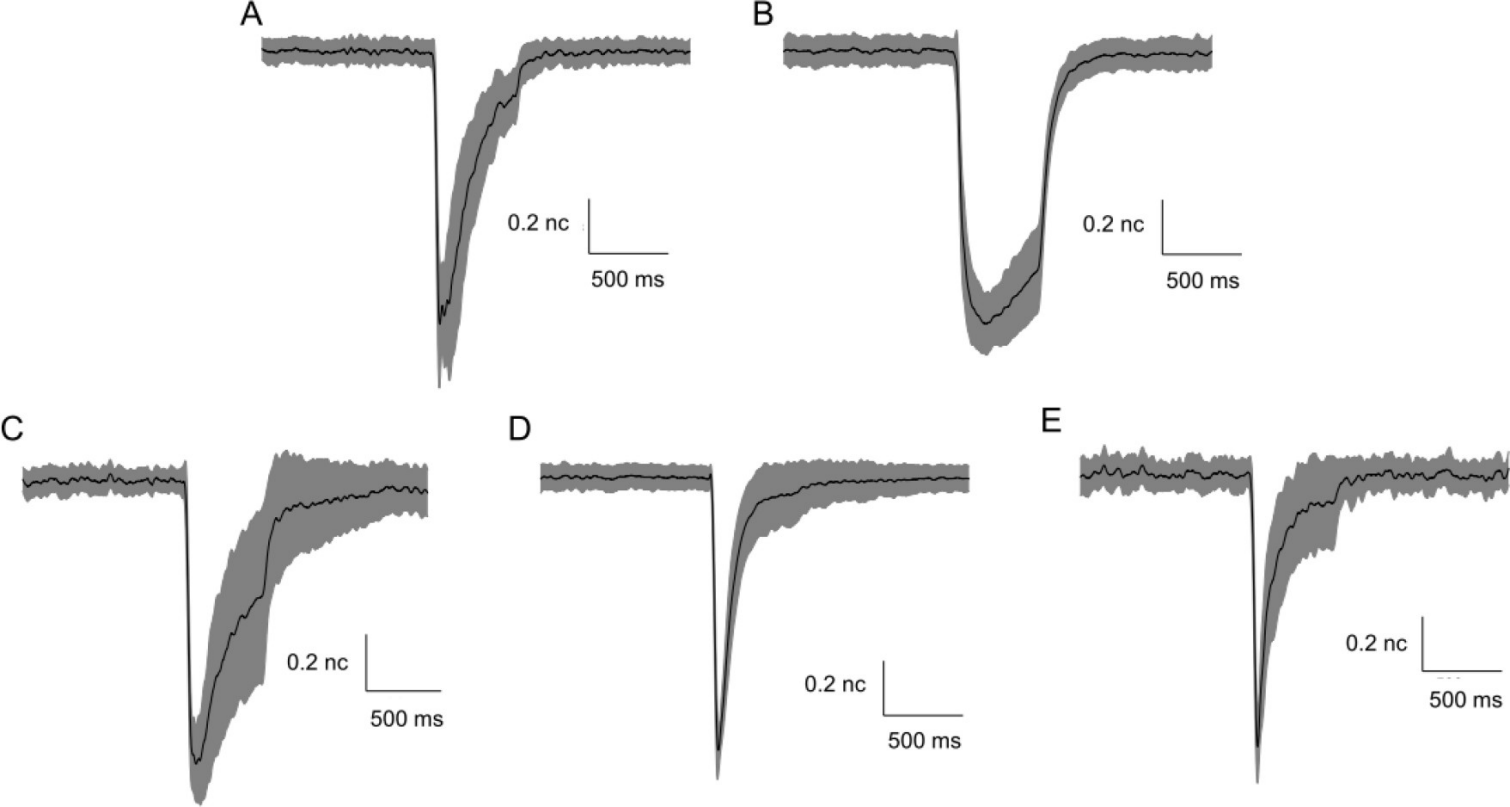
Normalized mean whole-cell currents from cotransfected E2117A and E2117D. Panels on top show normalized mean currents from cells transfected with either E2117A **(A)** or E2117D **(B)**. The panels on the bottom show normalized mean currents from cotransfection of E2117A and E2117D in different ratios: E2117A:E2117D::1:2 (**Panel C**), E2117A:E2117D::1:1 **(D)** and E2117A:E2117D::2:1 **(E)**. The mean current is the central black trace and the standard deviation in the data is the filled gray areas. Since these currents are normalized, the scale representing these values is labeled **“nc”** for normalized current. The inactivation profiles of the mean currents in these cotransfections seem to follow the profile of whichever channel is expected to be expressed more densely. Hence, E2117A:E2117D::1:2 appears more like E2117D and E2117A:E2117D::2:1 appears more like E2117A.

Are heteromers evident in macroscopic currents? We measured macroscopic currents by indenting cells with fire polished glass pipettes [1]. We varied the proportions of DNA for each of the homomers (E2117A:E2117D::1:2, 1:1 and 2:1). The kinetic features of whole-cell currents for E2117A and E2117D were reminiscent of those observed in cell-attached patches (**Fig 2A and 2B**), despite the change of the physical environment [2] between patch recordings versus whole-cell recording. The kinetics of the current from cotransfected cells depended on the ratio used for transfections (**Fig 6C and 6E**). For a 1:1 ratio, the mean current waveform (**Fig 6D**) was intermediate between that of E2117A and E2117D homomers suggesting that the channels were heteromers, But it took quantitative analysis to analyze whether the currents were merely the sum of two homomeric currents.

**Fig 7:**
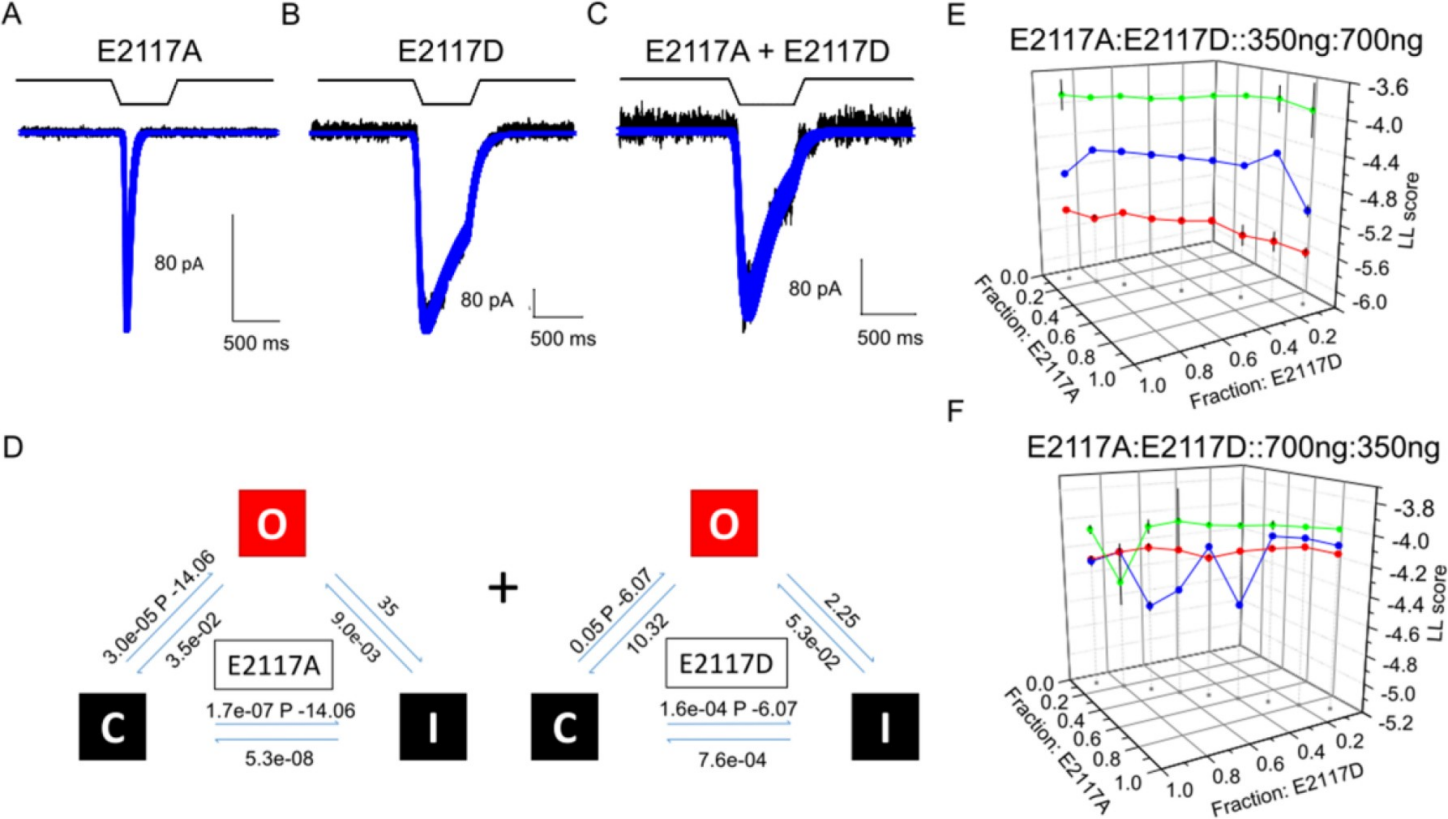
Modeling titration analysis of coexpressed E2117A and E2117D channels. These kinetic analyses examined whether whole-cell current from coexpressed, E2117A and E2117D, can be interpreted as arising from the sum of the currents from homomers. **(A)** and **(B)** show the current traces (in black) for the homomers. The fits (in blue) generated by models for the homomers currents are superimposed on the current traces. All kinetic models were executed in QuB. The models for macroscopic currents of homomers are in **(D)** with the summing operator “+”. A representative trace of the current from a cell that is coexpressing both channels is shown in **(C)** with the predicted current from the sum of independent homomers superimposed. **(E)** and **(F)** both show the log-likelihood (LL) values of the fits plotted against the fractions of E2117A and E2117D for a total of 300 channels. Each LL value is a measure of the congruence between the model-generated fits and the actual current trace from a patch-clamp experiment. Three data traces (red, blue and green) were used for modeling. **(E)** shows that the congruence between the model and experimental data is better when the fraction of E2117A channels is low and gets worse for higher fractions of E2117A. The modeling is in agreement with experimental data because transfecting with a ratio of E2117A:E2117D of 350ng:700ng **(E)** suggests that the cell would express fewer E2117A than E2117D. **(F)**, For a reversed transfection ratio (E2117A:E2117D::700ng:350ng), the congruence between the model and experimental data is better when the fraction of E2117A channels is high. Overall, these analyses show that the current in cotransfected cells, can be accounted for by the summation of the homomer currents and does not arise from a significant population of heteromers.

We constructed kinetic models of the homomers (**E2117A: Fig 7A and 7D; E2117D: Fig 7B and 7D**) and tested whether the observed cotransfection currents could be reproduced by a linear combination of the homomers (i.e. the current **I** = (α * i_E2117A_) + (β * i_E2117D_), where α and β are the fractions of each current type (β = 1-α). We optimized a series of models of ordered pairs of α and β, setting as the null hypothesis, that the combined current (**Fig 7C**) was produced by a sum of two monomeric populations. Comparing log-likelihoods across population ratios (**Fig 7E and 7F**) showed that the observed currents fit well with two independent homomeric populations rather than a significant heteromeric population.

### Heteromer formation between hPIEZO1 and hPIEZO2

**Fig 8:**
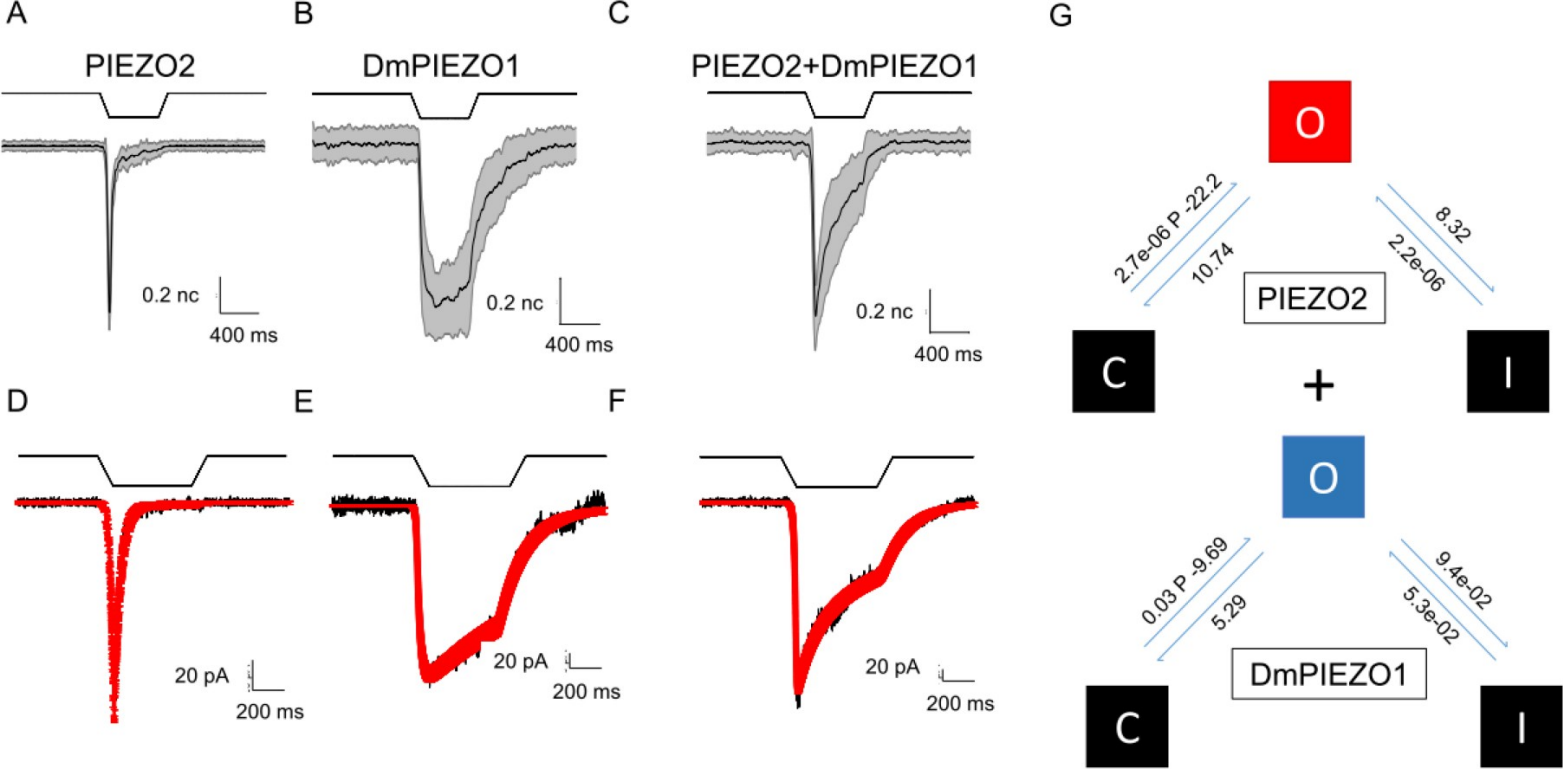
Kinetic analysis of current from hP1, hP2 cotransfections. Does coexpressing hP1 and hP2 lead to the formation of heteromers? To help differentiate between these two types of channels we used the double mutant form of PIEZO1 (PIEZO1-M2225R-R2456K), which has distinctly slower kinetics than wild-type channels. **(A)** and **(B)** show normalized and averaged traces for PIEZO2 and double-mutant PIEZO1, respectively. **(C)** shows normalized and averaged current from a cell expressing both channels. **(D)** Current from a PIEZO2-expressing cell superimposed with the fit generated by the model shown in the top part of **(G)**. **(E)** Current from a cell expressing the double-mutant form of PIEZO1 with a fit (red trace) superimposed; the model is displayed at the bottom of **(G). (F)** The current from a cell coexpressing both channels superimposed with a fit generated by summing the homomeric models for PIEZO2 and the double-mutant form of PIEZO1. This fit suggests that a simple sum of the homomeric models **(G)** can account for the observed kinetics and does not require heteromer activity.

We also examined whether heteromeric channel assemblies composed of human PIEZO1 (hP1) and PIEZO2 (hP2) subunits could give rise to currents with kinetics distinctly different from those composed of the sum of homomeric subunits [23]. To enhance the distinction between hP1 and hP2, we substituted hP1 with the double-mutant of hP1 (denoted DmPIEZO1; carrying mutations M2225R and R2456K) which inactivates very slowly [10]. We used a 1:2 ratio of hP1 to hP2 DNA (350ng DmPIEZO1: 700ng hP2). Whole-cell currents from cotransfected cells showed a fast component and a slow component. We analyzed these data again testing the null hypothesis that the observed currents were simply the sum of independently gated hP1 and hP2 populations. The observed current fit well assuming a sum of independent populations of homomers (**Fig 8**). We were unable to observe any features suggesting heteromer formation.

## Discussion

The PIEZO1 mutants we analyzed exhibited new unitary conductances and inactivation kinetics and are similar to those resulting from homologous mutations in mouse Piezo1 [15]. These mutants do not exhibit significant changes in cation selectivity (Li^+^ versus K^+^). The conservative E2117D substitution *increased* conductance while most others lowered it. E2117K produced no discernible current; this may be because E2117K adopts a stable inactivated state, much like the one caused by the effect of low pH on inactivation [24]. The correlation between modified inactivation rate and increased unitary conductance observed with E2117D is similar to that seen with the slow-inactivating mutant of *Shaker* channels [25] obtained by substituting a pore alanine to valine that also shows increased conductance and slowed C-type inactivation [26].

Kinetic modeling of E2117A, our fast-inactivating mutant, showed an increase in the slope sensitivity of activation. The slope sensitivity is a function of the free energy required for activation. In a simple model of a cylindrical channel that increases its peripheral radius upon opening, *ΔG = TΔA* [27, 28], where *T* is the local bilayer tension and *ΔA* is the change of in-plane area between the closed and open states [27]. It is unclear why some mutations, such as E2117A, increase the slope sensitivity, while others, such as the conservative substitution E2117D, have no effect. The observation that a particular residue may affect slope sensitivity implies that there are short-range interactions at the 2117 position during the opening process. The simple gating model above with *ΔG = TΔA* cannot distinguish between changes in *ΔA* and *T*. We know that the multiple channels cluster in discrete spatial domains [10], and the line tension of those domains could alter *T*, the local tension felt by the channels [29, 30]. Mutations might also alter line tension (energetics) of the domain by affecting the affinity of the channels for each other.

Cryo-electron microscopy shows that the channel is a trimer and the pore is central and formed at the interface of the carboxy-terminal domains of the three subunits [4, 6]. Specific C-terminal region residues were identified as central for ion permeation [31, 32]. The 2117 residue is not directly involved in subunit interactions but appears to modulate it (**Fig 1A)**. Our observation that co-expression of mutant subunits leads to the formation of heteromeric channels supports the model of these channels being trimers [4]. We tested E2117K and E2117D because they had the maximum difference in unitary conductance: 0 & 73 pS. The E2117K-E2117D combination is predicted to have only 3 observable conductance levels (KKD, KDD, DDD) because the E2117K trimer (KKK) is non-conducting and this was indeed what we observed. We also tested whether the two conducting mutants, E2117A and E2117D, could form heteromers. The combination of E2117A and E2117D should show four unique levels (AAA, DDD, ADD and AAD) arising from heteromers. Again, we observed four levels as predicted.

Coexpression of mutant channels resulted in single-channel currents with amplitudes intermediate to those of the homomers, and as expected of heteromers. Therefore, populations of heteromers indeed assemble from different subunits and can be detected by cell-attached patch recordings. The whole-cell currents resulting from coexpression showed intermediate inactivation rates and we modeled these currents using a linear combination of homomeric channel currents (**Fig 7**). This linear-combination model does not preclude the presence of heteromeric channels. Rather, it explains the whole-cell current using two predominant populations of channels that are observed in microscopy studies.

In a cell expressing more than one mRNA, homomers might result from expression of a given mRNA in a cellular domain where the newly formed channel subunits would be limited to self-association (homomers). Domains thus promote homomer formation, and consequently, there will be fewer interactions between unlike subunits of the coexpressed pair. Imaging cells with the two different PIEZO1 mutants, internally labelled with fluorescent probes [33], showed discrete homomeric domains and regions where channels were mixed (**S5 Fig**). Such segregation could easily translate into a whole-cell current in which homomer currents dominate the response and one that fits well to a linear-combination model. Furthermore, domains modulate the response of channel species by modifying the fraction of mean stress that reaches the channels from the stimulation site.

Currently, it is not possible to examine each heteromer species in isolation because we rely on coexpression to generate them, and there are no techniques available to force selective assembly of any given PIEZO heteromer. Consequently, it is not possible to obtain and incorporate a heteromer current signature into the whole-cell current modeling process. Our single-channel experiments indicate that heteromers do assemble; however, in the whole-cell mode homomer currents can mask those of the heteromers. Furthermore, the correlation we observe between imaging data and the linear-combination model strengthens the interpretation that homomer currents dominate because homomers self-associate in domains. Channel subunits occasionally fail to heteromerize despite sharing considerable sequence homology as shown by Bharill et al. [34]. The reasons for such failure have remained unclear, but we hypothesize that this phenomenon may be the result of domain-localized mRNA translation.

The C-terminal residues form the ion-conducting pore [15] and determine the rate of inactivation of PIEZO channels. More recent studies indicate that the C-terminal is involved in Ras signaling [35], but the gamut of processes it is involved in continues to expand. We show here that altering a single C-terminal residue (E2117) alters the channel’s biophysical properties by heteromeric assembly with consequences to the mechanism of action of PIEZO channels *in situ*. Thus far, no disease-causing mutations at this site have been reported. However, a linear-combination-like response was observed in chondrocytes and articular cartilage, where PIEZO1/ PIEZO2 coexpression produced a new kinetic response [23]. We also find that when mutant channels are coexpressed, the cellular response to mechanical stress results from a combination of heteromers and homomers, and the latter frequently reside in domains where they are prone to self-association and hence dominate the response. Proteins interacting with PIEZO (like tentonin3/TMEM150c [36]), also seem to prolong inactivation, which is a recurring theme common with several mutations of the C-terminus. In conclusion, these studies are vital to gain a better understanding of how PIEZO channels and their aforementioned interacting partners operate in cells.

## Materials and methods

### Cloning

A segment of the PIEZO1 gene [10] between the Nhe1 and BamH1 restriction sites, 9from 3960 to 7602) was excised and DNA was inserted into the plasmid pIRES2 EGFP by ligation. Site-directed mutagenesis on this DNA using Prime Star GXL polymerase (Clontech/Takara) with 50 ng of template DNA and 0.2 μM each of forward and reverse primers in a 2-step PCR reaction: 98°C for 15 seconds and 68°C for 9 minutes, 20 cycles. Primers are listed below. The PCR product was treated with Dpn1 and column purified before transforming into Stellar competent cells (Clontech/Takara) and plated onto plates containing kanamycin. Clones were sequenced to ensure the presence of the desired mutation. DNA was amplified using a Sbf forward primer and Xho reverse primer.

To make the full length clone, the fragment bearing the mutation was excised by treatment with Sbf1 and Xho1 restriction sites, then gel purified (1% agarose gel) and further purified by with Nucleospin (Invitrogen) according to manufactures specification. The plasmid DNA (50–75 ng) bearing PIEZO1 was prepared in a similar manner. The two DNAs were assembled by an Infusion reaction (Clontech/Takara) in a 5 μl reaction for 15 minutes and transformed into Stellar competent cells (Clontech/Takara). Clones were verified by sequencing.

Primers:

HP1E2117DFwd–GTGCCGTTCCTGGTGGATCTGCGGGCAGTGATGG

HP1E2117Drev–CCATCACTGCCCGCAGATCCACCAGGAACGGCAC

HP1E2117AFwd–GTGCCGTTCCTGGTGGCGCTGCGGGCAGTGATG

HP1E2117Arev–CATCACTGCCCGCAGCGCCACCAGGAACGGCAC

InfHP1SbfF–TCACACAGGAGCTCCTGCAGGG

InFIEHP1Xho1R –CCGAGGTGCCCTCGAGCAGGCTG

### Electrophysiology

For cell-attached recordings, the pipette solution usually contained (in mM) 160 KCl, 0.25 EGTA and 10 HEPES, pH 7.3 (adjusted with KOH). The bath solution contained (in mM) 168 KCl, 1 MgCl_2_, 1 CaCl_2_, 10 HEPES, at pH 7.3 (adjusted with KOH).

HEK-293 cells were transfected with 200–500 ng of cDNA using TransIT-293 reagent (Mirus) according to the manufacturer’s protocol, and tested 24-48h later. The dosing ratio used for formation of E2117K and E2117D heteromers was 2:1, 1:1 or 1:2, and the total amount of transfected cDNA was ~500 ng.

Cell-attached patches were mechanically stimulated by applying suction with an ALA pressure clamp (HSFC-1, ALA Scientific instruments) controlled by QuBIO software[37]. Whole-cell mechanical stimulation indented the cell with a fire-polished glass pipette (diameter of 2–4 μm) positioned at an angle of 30^o^ with respect to the cover glass. The probe was coarsely positioned ~20 μm above the cell with a MP-285 manipulator (Sutter Instruments Co.), and from that position, the probe moved up and down by 1 micron with a trapezoidal waveform using a piezoelectric positioner (P-280.20 XYZ NanoPositioner, Physik Instrumente). The indentation depth was controlled using LabVIEW software providing 40 nm resolution. The probe velocity was constant at 0.15 μm/ms during transitions, and the stimulus was held constant for 300 ms. Currents were recorded at room temperature using an Axopatch 200B amplifier (Axon Instruments) sampled at 10 kHz and filtered at 1 kHz. Data acquisition and stimulation were controlled by QUBIO software. Modeling whole-cell current as a linear combination was performed in QuB Express using the MacRates plugin. The indentation stimulus was normalized (stimulus value of “1” is completely on) and used to construct models of homomer currents. These homomer models were summed to perform the linear combination modeling and values for the total number of channels was provided as input for the process. The number of channels assigned to each model was determined by fractional values: α and β, such that α = 1 –β.

## Supporting information

S1 Fig Two mutations of E2117 produce lower unitary conductance. We replaced the glutamic acid with either cysteine **(A)** or serine **(B)** and measured the single channel currents. Cell-attached patch recordings at -100 mV in a potassium bath. The amplitude histogram shows that both homomeric channels have a low conductance similar to E2117A. For E2117C, the single channel current was 3.3 ± 0.1 pA and for E2117S, 3.7± 0.2 pA.
(TIF)

S2 Fig First order characterization of ion selectivity through E2117A and E2117D. **(A)** is the current for E2117A with either LiCl or K-gluconate in the pipette compared to KCl. Cell-attached patch recordings performed at -100 mV in a high potassium bath. **(B)** is from E2117D using the same pipette solutions. Li^+^ used to evaluate selectivity. In wild-type, K^+^ conductivity is twice that of Li^+^. This ratio was maintained by both mutated channels and was the same as the wild-type indicating that permeation selectivity was not significantly altered **((C) and (D))**. We tested the cationic selectivity by recording with K^+^- gluconate vs KCl and demonstrated that the currents and reversal potentials were similar. The data for E2117A summarized in **(C)** and for E2117D in **(D)**.
(TIF)

S3 Fig Standard deviation errors to the kinetic rate constants modeled in Fig 3D. (TIF)

S4 Fig Log-likelihood values for titration analysis of E2117A and E2117D cotransfections. **Fig 7** shows titration of various fractions of the two mutants performed with a total of 300 channels. Those same data were reanalyzed using either 200 (two top panels) or 400 (two bottom panels) channels to observe the dependence of the fit on channel number density.
(TIF)

S5 Fig Spatial distribution of heteromeric and homomeric channels in a cotransfected cell. **Left Panels**—E2117D internally labeled with EGFP and E2117A labeled with mCherry. The channels’ functional behavior in a patch is unaffected by the presence of the labels [33]. Top panel is a single cell and bottom panel is a cluster of cells. Imaging Z-stacks Right Panels- Expression of wild-type channels internally labeled with EGFP or mCherry. Top panel is a single cell and the lower image is a cluster of cells. Images were acquired with a Visitech VTI-iSIM on a Nikon TE2000 microscope excited at 488nm and 568nm captured with 48 z-intervals of 200 nm. Regions showing overlap between the two mutant channels are yellow and represents heteromeric domains; regions where channels segregate into discrete domains are either green or red. The reason for inhomogeneity is unclear but the phenomenon of lack of coassembly among subunits with sequence similarity has been observed before [34]. Wild-type channels show similar expression inhomogeneity indicating that channel properties cannot account for the distribution of channels. The bar is scaled to 1 μm.
(TIF)
