# Supplementary figures and images for: Functional analyses of heteromeric human PIEZO1 Channels
Source: PLoS One. 2018 Nov 21;13(11):e0207309. doi: 10.1371/journal.pone.0207309 (PMC6248943; doi:10.1371/journal.pone.0207309)

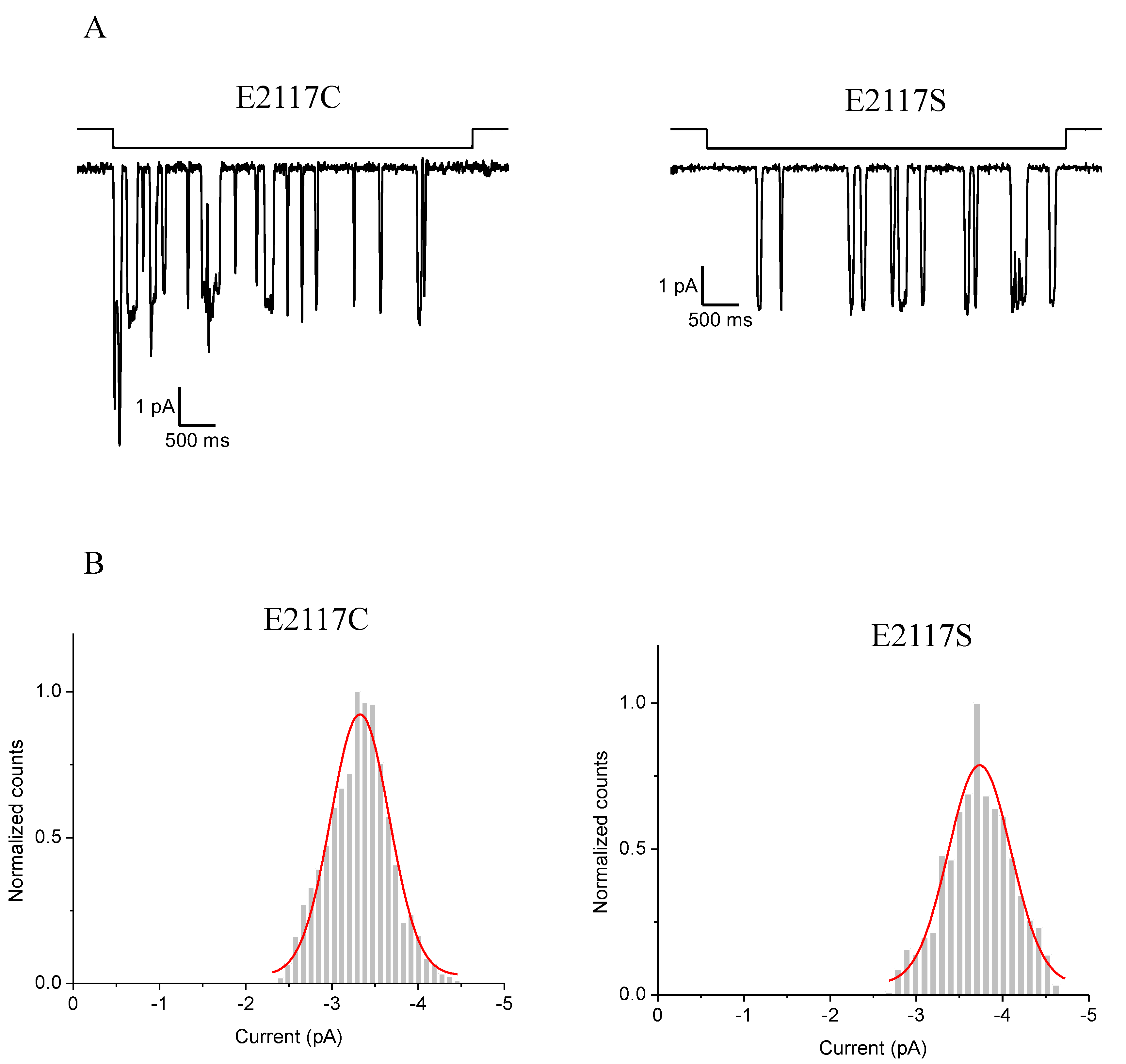

Supplement: S1 Fig — We replaced the glutamic acid with either cysteine (A) or serine (B) and measured the single channel currents. Cell-attached patch recordings at -100 mV in a potassium bath. The amplitude histogram shows that both homomeric channels have a low conductance similar to E2117A. For E2117C, the single channel current was 3.3 ± 0.1 pA and for E2117S, 3.7± 0.2 pA. (TIF) [file pone.0207309.s001.tif]

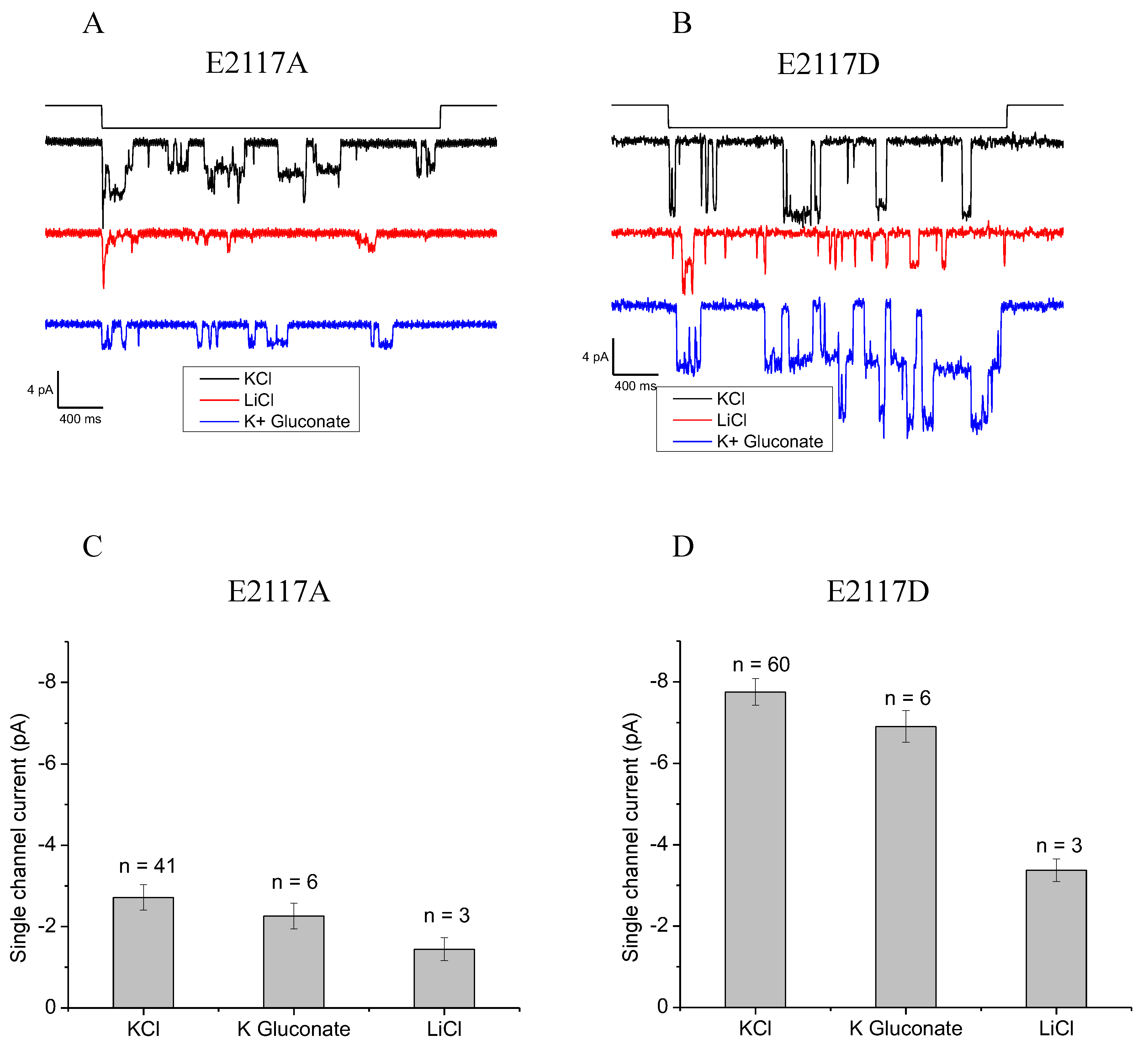

Supplement: S2 Fig — (A) is the current for E2117A with either LiCl or K-gluconate in the pipette compared to KCl. Cell-attached patch recordings performed at -100 mV in a high potassium bath. (B) is from E2117D using the same pipette solutions. Li+ used to evaluate selectivity. In wild-type, K+ conductivity is twice that of Li+. This ratio was maintained by both mutated channels and was the same as the wild-type indicating that permeation selectivity was not significantly altered ((C) and (D)). We tested the cationic selectivity by recording with K+- gluconate vs KCl and demonstrated that the currents and reversal potentials were similar. The data for E2117A summarized in (C) and for E2117D in (D). (TIF) [file pone.0207309.s002.tif]

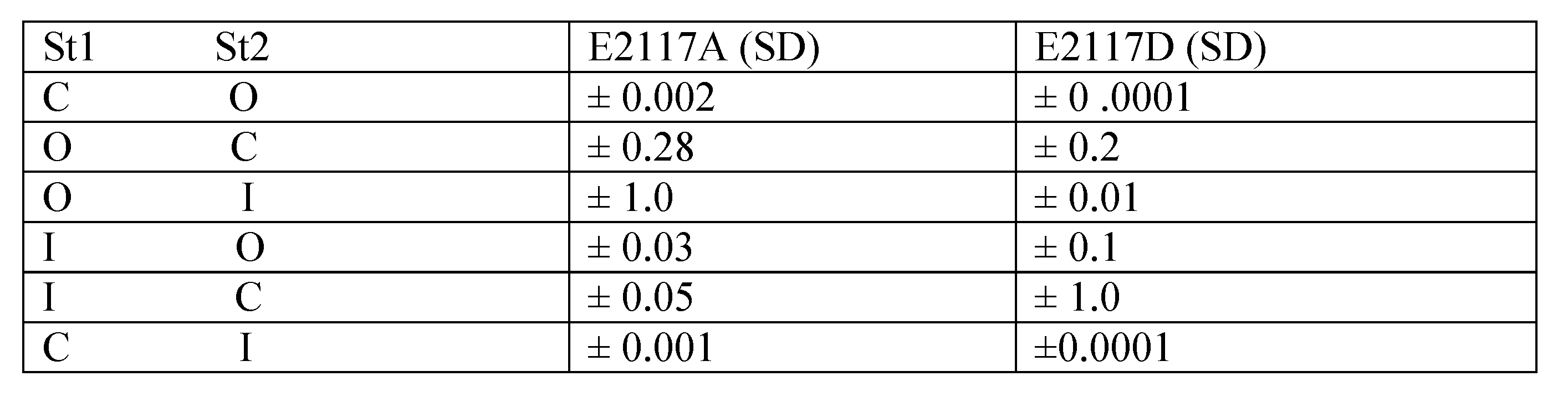

Supplement: S3 Fig — (TIF) [file pone.0207309.s003.tif]

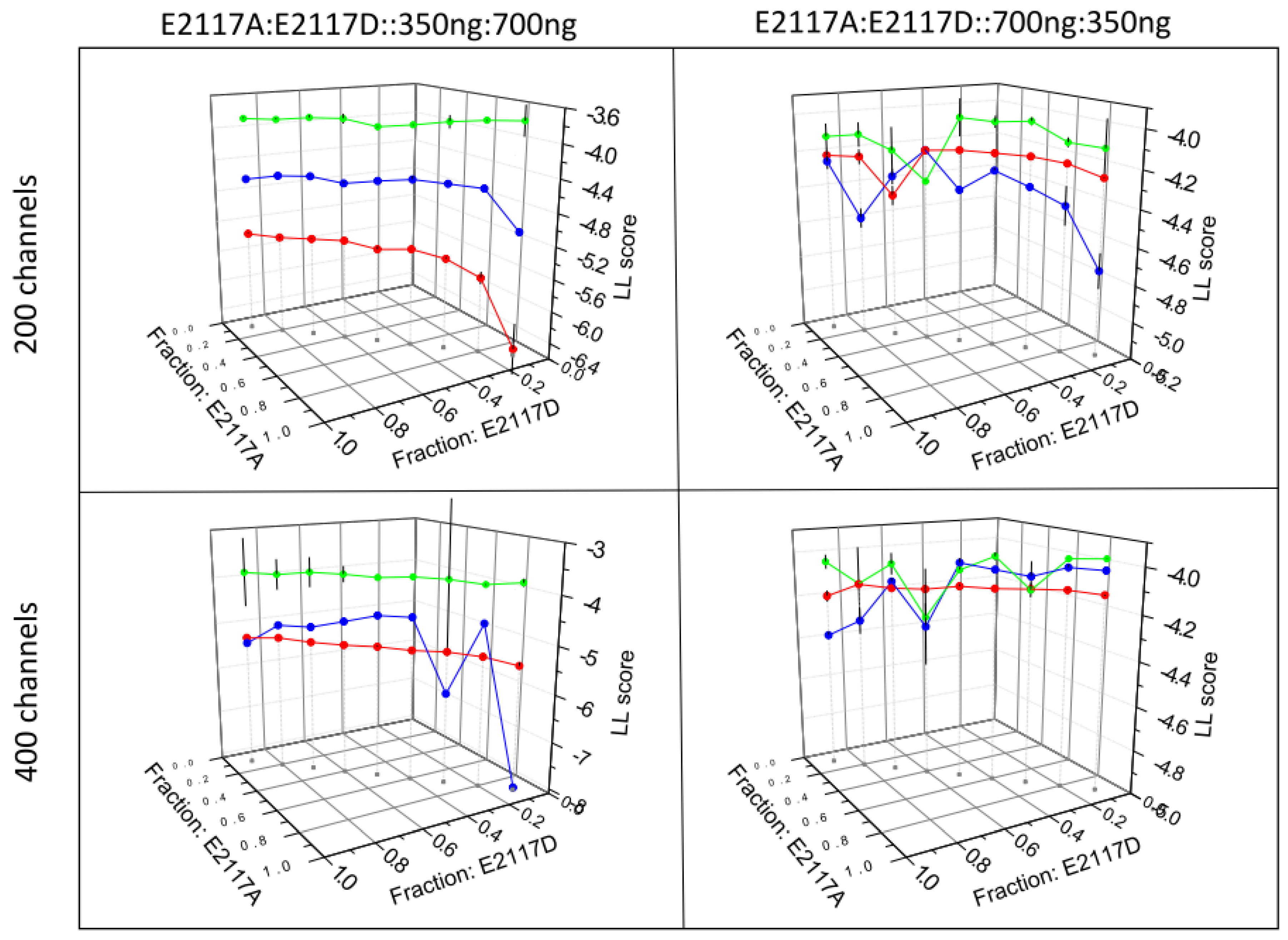

Supplement: S4 Fig — Fig 7 shows titration of various fractions of the two mutants performed with a total of 300 channels. Those same data were reanalyzed using either 200 (two top panels) or 400 (two bottom panels) channels to observe the dependence of the fit on channel number density. (TIF) [file pone.0207309.s004.tif]

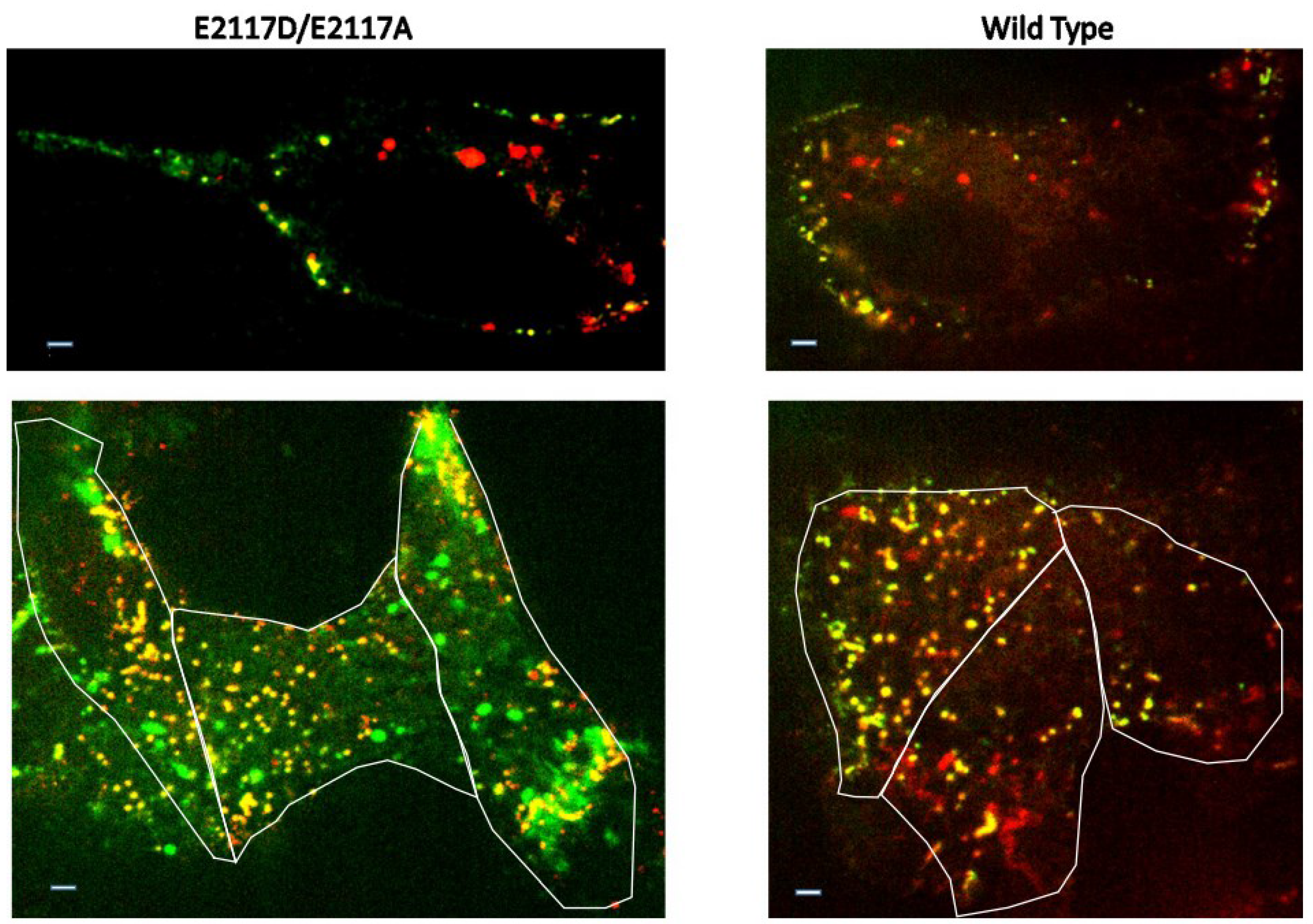

Supplement: S5 Fig — Left Panels—E2117D internally labeled with EGFP and E2117A labeled with mCherry. The channels’ functional behavior in a patch is unaffected by the presence of the labels [33]. Top panel is a single cell and bottom panel is a cluster of cells. Imaging Z-stacks Right Panels- Expression of wild-type channels internally labeled with EGFP or mCherry. Top panel is a single cell and the lower image is a cluster of cells. Images were acquired with a Visitech VTI-iSIM on a Nikon TE2000 microscope excited at 488nm and 568nm captured with 48 z-intervals of 200 nm. Regions showing overlap between the two mutant channels are yellow and represents heteromeric domains; regions where channels segregate into discrete domains are either green or red. The reason for inhomogeneity is unclear but the phenomenon of lack of coassembly among subunits with sequence similarity has been observed before [34]. Wild-type channels show similar expression inhomogeneity indicating that channel properties cannot account for the distribution of channels. The bar is scaled to 1 μm. (TIF) [file pone.0207309.s005.tif]
